# Occupational stress and burnout among intensive care unit nurses during the pandemic: A prospective longitudinal study of nurses in COVID and non-COVID units

**DOI:** 10.3389/fpsyt.2023.1129268

**Published:** 2023-03-13

**Authors:** Pratima Saravanan, Tariq Nisar, Qian Zhang, Faisal Masud, Farzan Sasangohar

**Affiliations:** ^1^Center for Health Data Science and Analytics, Houston Methodist, Houston, TX, United States; ^2^Department of Anesthesiology and Critical Care, Houston Methodist DeBakey Heart and Vascular Center, Houston Methodist, Houston, TX, United States; ^3^Department of Industrial and Systems Engineering, Texas A&M College of Engineering, Texas A&M University, College Station, TX, United States

**Keywords:** critical care, physiological variable, nursing, naturalistic study, burnout, stress

## Abstract

**Background:**

Intensive care unit (ICU) nurses are highly prone to occupational stress and burnout, affecting their physical and mental health. The occurrence of the pandemic and related events increased nurses’ workload and further exacerbated their stress and burnout. This work investigates occupational stress and burnout experienced by ICU nurses working with COVID and non-COVID patients.

**Method:**

A prospective longitudinal mixed-methods study was conducted with a cohort of ICU nurses working in medical ICU (COVID unit; *n* = 14) and cardiovascular ICU (non-COVID unit; *n* = 5). Each participant was followed for six 12-h shifts. Data on occupational stress and burnout prevalence were collected using validated questionnaires. Physiological indices of stress were collected using wrist-worn wearable technologies. Participants elaborated on the causes of stress experienced each shift by completing open-ended questions. Data were analyzed using statistical and qualitative methods.

**Results:**

Participants caring for COVID patients at the COVID unit were 3.71 times more likely to experience stress (*p* < 0.001) in comparison to non-COVID unit participants. No differences in stress levels were found when the same participants worked with COVID and non-COVID patients at different shifts (*p* = 0.58) at the COVID unit. The cohorts expressed similar contributors to stress, based in communication tasks, patient acuity, clinical procedures, admission processes, proning, labs, and assisting coworkers.

**Conclusion:**

Nurses in COVID units, irrespective of whether they care for a COVID patient, experience occupational stress and burnout.

## 1. Introduction

Nurses may experience occupational stress when the needs and demands of their work are beyond their abilities and available resources, resulting in emotional and physical overload (1, 2). Prolonged exposure to stress may lead to “burnout,” a psychological phenomenon characterized by a decline in physical and emotional wellbeing, resulting in diminished self-appreciation and development of cynicism toward patients and coworkers (3, 4). The prevalence and causes of occupational stress and burnout among nurses have been studied (5, 6), including an extensive and recent (albeit pre-COVID-19) gap analysis on understudied factors (7); however, the occurrence of the COVID-19 pandemic instigated an unprecedented burden on nurses globally (8–11). Prior to COVID-19, as many as 40% of nurses in hospitals and nursing homes experienced high levels of stress and burnout (12). The increased workload and working hours, and frustration at negative outcomes despite the extra effort during the pandemic, have escalated stress levels (13, 14). Intensive care unit (ICU) nurses were disproportionately affected by such higher levels of stress, burnout, insomnia, and anxiety due to exceptionally high exposure to patient mortality (15, 16).

Several studies investigated the impact of the COVID-19 pandemic and related events on nurses’ mental and physical health. Negative effects of the pandemic to nurses’ mental wellbeing are not limited to those who cared for COVID patients or the nursing profession in general. For example, Yoon et al. (17) documented the increased stress among healthcare providers due to the “spillover” or the indirect effects of COVID-19 on the vulnerable non-COVID patients resulting in treatment disparities toward chronically ill and mental health patients. Furthermore, studies have supported that nurses who cared for non-COVID patients experienced higher levels of anxiety, PTSD symptoms, neuroticism, and poor coping strategies compared to the COVID unit nurses (18–20). Similarly, Doo et al. (21) found that the nurses in COVID units working with COVID-suspected patients suffered significantly higher anxiety, depression, and low resilience than nurses working with COVID-positive and non-COVID patients due to the increased uncertainty of infection risk and insufficiently protective equipment and environment. Studies focusing on burnout, insomnia, anxiety, depression, and unfinished patient care found that there was no significant difference in occupational stress between COVID and non-COVID nurse groups (16, 22, 23).

Despite this evidence suggesting the globally negative impact of the pandemic on nurses, more rigorous unit-level comparison is warranted. Most of these studies employed online surveys or questionnaires (18–21). While self-reported measures allow timely and remote data collection to document the psychological impact of the pandemic on the nursing profession (24), such methods are inherently prone to selection biases (non-response bias and self-selection bias) (25) and recall bias (26). To our knowledge, differences in occupational stress between nurses working in COVID and non-COVID ICUs have not been investigated using objective methods such as those using physiological data. Furthermore, most of these studies are not ICU-specific (17–20). In addition, recent reviews of the literature on the impact of the pandemic on nurses’ occupational stress and related factors (15, 27) show that most studies were conducted in the southern parts of Asia and Europe, and hence, some variance may exist across settings and the results may not be generalizable.

The use of physiological indices such as heart rate (HR), skin temperature (ST), and electrodermal activity (EDA) collected from wearable sensors has shown promise in recent studies that investigated occupational stress (28). HR or pulse rate is a widely used measure in stress research as HR increases significantly during stressful events (29, 30). Similarly, core body temperature increases during periods of stress and anxiety; studies have validated wrist ST as an indicator of stress (31, 32). EDA is the skin conductance of an individual and is influenced by the surface sweat glands. Phasic EDA—the acute, time-varying spikes in skin conductance level—significantly increases during states of high emotional arousal and stress (33, 34). Though a few studies used physiological indices to measure occupational stress of ICU nurses with promising results (31, 35), the studies were cross-sectional rather than comparative.

To address these gaps, the objective of this research was to compare the occupational stress and burnout levels of ICU nurses caring for COVID-19 and non-COVID-19 patients using a combination of physiological and self-reported metrics in a large health system in the Southwestern United States.

## 2. Materials and methods

A prospective longitudinal mixed-methods study was conducted with a cohort of ICU nurses working in two ICUs. This research complied with the Declaration of Helsinki and was approved by the Houston Methodist Research Institute Institutional Review Board (PRO00031545). Written informed consent was obtained from each participant at the start of the study.

### 2.1. Settings and participants

Participants of this study were registered nurses (RNs) working in two 36-bed ICUs at Houston Methodist Hospital, a large metropolitan tertiary care hospital in the Greater Houston area. Recruitment emails were sent to all RNs in the Medical ICU (MICU) and Cardiovascular ICU (CVICU). Initially, 21 RNs were recruited (*n* = 15 from MICU and *n* = 6 from CVICU); however, only 19 nurses completed the study. Participants were a combination of day and night shift nurses working 12-h shifts. On average, participants were 33-years old (SD = 10.3 years) and had 49.5 months of experience working at their current ICU (SD = 65 months) and 71.68 months of experience working as a registered nurse (SD = 90 months). The demographics of the participants are shown in Table 1. MICU participants cared for both COVID and non-COVID patients, whereas CVICU participants cared primarily for non-COVID cardiovascular patients.

**Table 1:** Participant demographics.

	MICU	CVICU
Gender, n (%)		
Female	12 (85.72)	5 (100)
Male	2 (14.28)	0 (0)
Shift, n (%)		
Day	9 (64.28)	5 (100)
Night	5 (35.72)	0 (0)
Race/Ethnicity, n (%)		
Hispanic or Latin	3 (21.42)	1 (20)
White	5 (35.71)	3 (60)
Asian	5 (35.71)	0 (0)
Black or African American	1 (7.1)	0 (0)
American Indian/Alaska Native	0 (0)	1 (20)
Marital status, n (%)		
Single, never married	6 (42.85)	3 (60)
Married or domestic relationship	8 (57.15)	2 (40)
Age in years, *M* (*SD*)	32.85 (9.75)	31.2 (12.44)
Months of experience at current unit, *M* (*SD*)	45.62 (57.48)	57 (86.16)
Months of experience as a registered nurse, *M* (*SD*)	79.15 (49.82)	108.6 (144.87)

### 2.2. Data collection

Data collection was conducted at the intersection of Delta and Omicron COVID-19 variants’ predominant period (early November of 2021 to the end of January 2022) (36). A total of 654 patients were admitted to MICU and CVICU combined during the 3 months of data collection (226 patients in November 2021, 230 patients in December 2021, and 227 patients in January 2022). Out of the 654 patients, 148 were COVID-positive patients admitted in the MICU (23, 51, and 84 patients admitted in the months of November, December, and January, respectively).

Data were collected from each participant for six 12-h working shifts, resulting in a total of 114 shifts and over 82,000 min of data. At the beginning of the first 12-h shift, participants completed demographic and pre-study questionnaires that included: Maslach Burnout Inventory for Medical Professionals (MBI-MP), a 22-item questionnaire focusing on emotional exhaustion, depersonalization, and personal achievements (37); Generalized Anxiety Disorder scale (GAD-7), a 7-item questionnaire that categorizes the anxiety severity (38); Perceived Stress Scale (PSS), a 10-item questionnaire that quantifies and categorizes perceived stress levels (39); and the Occupational Fatigue Exhaustion/Recovery scale (OFER), a 15-item questionnaire focusing on chronic fatigue, acute fatigue, and inter-shit recovery of participants (40).

The Empatica E4 (41), a wearable, lightweight, non-invasive, wristwatch-like device was used to collect real-time physiological data for all six shifts of data collection. The E4 device comprises an electrode that continuously records EDA, a temperature sensor that records ST, a photoplethysmography to record blood volume pulse from which heart rate (HR) and inter-beat interval (IBI) were derived, and a three-axis accelerometer. The E4 sensors are considered medical-grade and prior studies have used it for stress detection (35, 42–45). A fully charged E4 was given to the participants at the start of each shift and returned at the end of shift. The recorded physiological data were exported to a secure cloud platform, E4 manager, where continuous time-series data for each physiological variable were generated in .csv format. The sampling rate for the generated HR data was 1 Hz, and ST and EDA were sampled at 4HZ. The participants also wore two Axivity sensors, one on lap and the other on abdomen to assess the physical activities. The E4 also comprises a 3-axis accelerometer that captures the hand motions of the participants. However, this study documents only the physiological data from the E4, and the physical activity data will be reported elsewhere. The computation of phasic EDA from raw EDA, stress index (SI), and energy expenditure (EE) from blood volume pulse are detailed in the data processing section.

At the end of each shift, a post-shift questionnaire asked participants to rate their perceived shift difficulty/stress level and to answer two open-ended questions: (1) Please comment on contributors to your stress during the current shift and (2) Were there any differences in roles/activities between the current shift and previous shifts that might have contributed to your stress.

### 2.3. Data processing

Raw physiological data from the E4 device were processed for further analysis. Cut-off values were defined for HR and ST to remove artifacts. In line with Ahmadi et al. (35), any HR value above 200 bpm and ST values above 45°C were removed, and the averages per minute was computed. A Python script incorporating the Ledapy package (46) was used to correct artifacts and segregate phasic and tonic components of EDA. SciPy, a signal processing package in Python, was employed to extract the amplitude of phasic EDA (47), and the averages of peak amplitude per minute were calculated.

Kubios V3.3.1 was used to calculate SI and EE at 1-min intervals from the IBI signals. SI is the square root of Baevsky’s stress index (48) and relates to the intensity of sympathetic cardiac function, and has been used as an indicator of stress (35, 49). Baevsky’s stress index between 50 and 150 c.u is considered as a normal stress zone, and a value over 150 c.u represents a high stress zone (48). Hence, an SI value less than 12.2 c.u is considered as a low stress zone, a value over 12.2 c.u. as a high stress zone (50). Past studies have used SI to study cardiac surgery residents’ psychological and psycho-emotional stress levels (51) and assess a training program for adult psychosomatic self-regulation (52). Kubios computes EE based on the participant’s gender, HR, weight, and age (53). The processed physiological data—HR (beats/min), ST (°C), phasic EDA (μS), SI (c.u.), and EE (kcal/min)—were used in the analysis.

### 2.4. Data analysis

Statistical analysis was employed to compare the questionnaire responses and physiological variables among nurses working at MICU and CVICU units. Qualitative analysis of open-ended responses collected at the end of each shift was performed to achieve contextualized understanding of the factors that instill stress and feeling of burnout among nurses.

#### 2.4.1. Statistical analysis

Measures of central tendency are summarized, and descriptive characteristics are reported, with means and standard deviations (SD) for continuous variables and proportions for categorical variables. Histograms were plotted, and Shapiro–Wilk and Anderson–Darling tests of normality were used to identify the distribution of continuous measures and outcomes.

**Figure 1:**
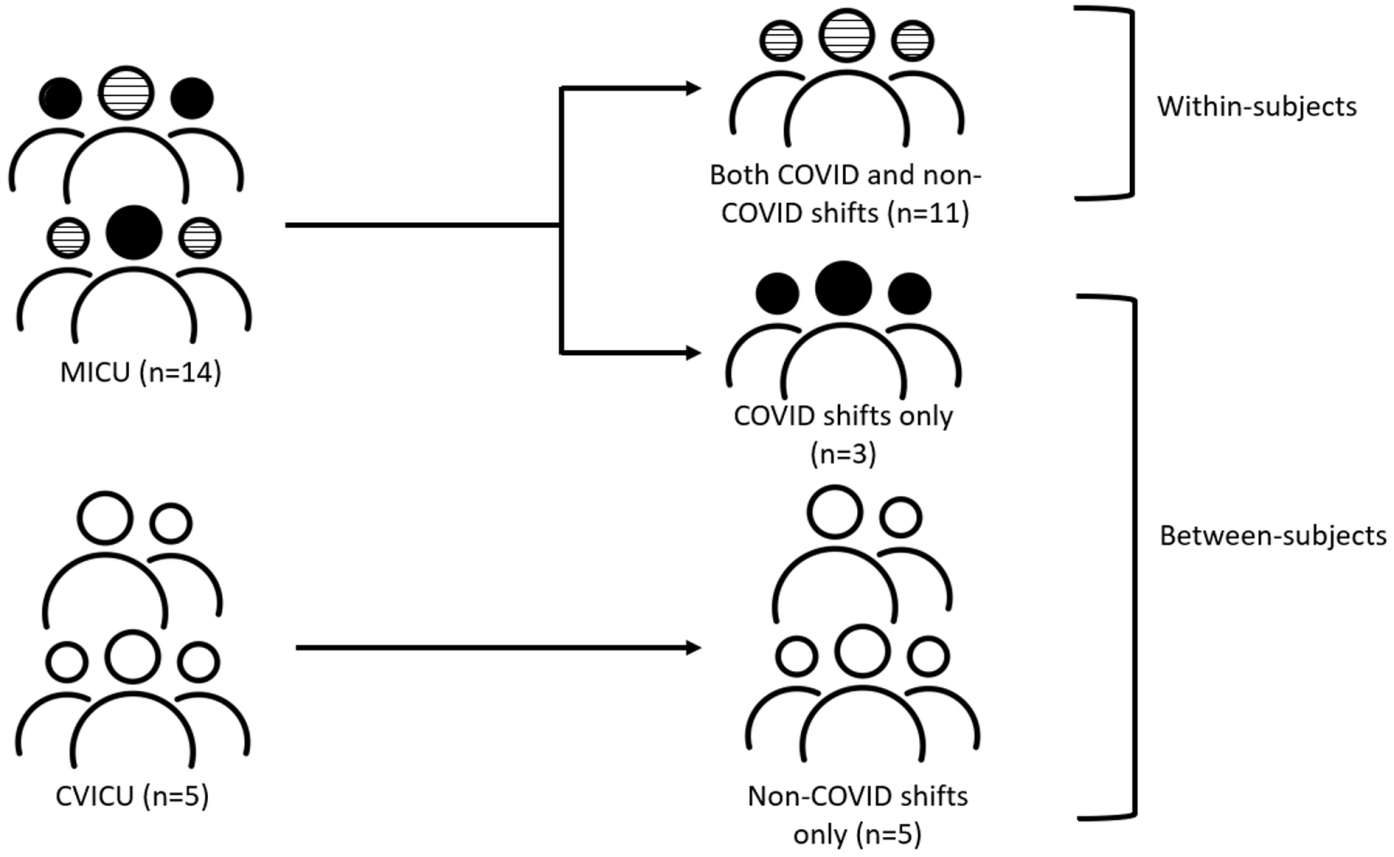
Statistical study design to compare stress levels in participants.

Between- and within-subject tests were performed to determine the differences in stress levels among participants caring for COVID and non-COVID patients (see Figure 1). The between-subject test was conducted to compare the stress levels of participants who worked only COVID shifts with participants who worked only non-COVID shifts during all six data collection shifts. The within-subject test was used to analyze the stress levels of the participants who worked both COVID and non-COVID shifts. Differences in stress levels were determined using univariable and multivariable logistic regression models using the generalized estimating equation (GEE) and random effect models accounting for repeated measurements. The outcome variable was stress levels, classified as 1 (high stress zone) vs. 0 (normal and low stress zones). The primary independent variable was COVID and non-COVID shiftwork. Based on prior literature on the variables related to burnout and occupational stress, the model was adjusted for participants’ demographics (age, number of children, and years of nursing experience in the ICU) (7), physiological variables (HR, phasic EDA, ST, EE) (28–35), and emotional exhaustion (54).

Statistically significant covariates from the univariable model along with lower Akaike Information Criterion (AIC) values were included in the multivariable model. Two-sided alpha of 0.05 was used to determine statistical significance. We report odds ratio (OR), 95% CI, and degrees of freedom (DF). All statistical analyses were performed using R statistical software version 4.1.1 (55).

#### 2.4.2. Qualitative analysis

The qualitative data obtained from the participants’ open-ended responses in the post-shift questionnaires were analyzed for COVID (MICU) and non-COVID (CVICU) units separately to identify the factors inducing stress in participants working at the respective units. The open-ended responses were coded by two authors (PS and QZ) experienced in qualitative coding. A codebook was developed using open and axial coding, and inductive thematic analysis was employed to identify new emergent themes (56). Any disagreements during the coding process were resolved by collective discussion of the coders until a thorough consensus was reached.

## 3. Results

### 3.1. Statistical findings

Table 2 displays the pre-study questionnaire and physiological variables of participants. Pre-study questionnaire data showed that on average, MICU participants experienced high emotional exhaustion and low personal achievement, whereas CVICU participants experienced moderate emotional exhaustion and high personal achievement. Both groups experienced moderate level of depersonalization. MICU participants had mild anxiety and CVICU participants showed minimal level of anxiety. Both groups displayed moderate levels of perceived stress. Though both groups experienced similar acute fatigue, MICU participants showed, on average, higher chronic fatigue and low inter-shift recovery when compared with CVICU participants.

**Table 2:** Descriptive statistics of pre-study questionnaire and physiological variables of participants in MICU and CVICU.

	MICU (COVID unit)	CVICU (non-COVID)
Pre-study questionnaire variables		
MBI-MP		
Emotional exhaustion, *M* (*SD*), category	27.0 (9.66), high	24.6 (8.65), moderate
Depersonalization, *M* (*SD*), category	9.0 (5.99), moderate	8.4 (5.32), moderate
Personal achievement, *M* (*SD*), category	35.23 (7.25), moderate	40.0 (3.39), high
GAD-7		
Anxiety severity, *M* (*SD*), category	6.23 (4.21), mild	3.4 (2.88), minimal
PSS		
Perceived stress, *M* (*SD*), category	18.38 (3.25), moderate	14.8 (4.55), moderate
OFER		
Acute fatigue, *M* (*SD*)	62.69 (23.95)	63.0 (18.23)
Chronic fatigue, *M* (*SD*)	46.54 (21.35)	34.0 (20.74)
Inter-shift recovery *M* (*SD*)	51.54 (21.45)	55.0 (18.71)
Physiological variables		
Stress index, SI (c.u.), *M* (*SD*)	12.55 (3.10)	12.81 (2.88)
Heart rate, HR (bpm), *M* (*SD*)	69.95 (8.91)	72.55 (8.37)
Skin temp, ST (°C), *M* (*SD*)	33.26 (1.38)	32.83 (1.51)
Phasic electrodermal activity, phasic EDA (μS), *M* (*SD*)	0.15 (0.29)	0.30 (0.66)
Energy expenditure, EE (kcal/min), *M* (*SD*)	1.05 (1.10)	1.00 (1.19)

MBI-MP, Maslach Burnout inventory for medical professionals; GAD-7, generalized anxiety disorder-7; PSS, perceived stress scale; OFER, occupational fatigue exhaustion recovery.

Physiological data showed that on average, both groups have high stress levels (>12.2 c.u.), with CVICU participants showing slightly higher SI than MICU participants. CVICU participants also exhibited high HR and phasic EDA compared to their MICU counterparts. Both groups showed similar ST and EE, with CVICU participants showing slightly higher ST and MICU participants exhibiting slightly higher EE.

Table 3 shows the percentage of time participants spent at high, normal, and low stress levels throughout their shifts. Since MICU comprised both COVID and non-COVID patients, the stress levels were distinguished based on the shift (COVID or non-COVID). CVICU participants spent the most time in high stress levels (54.3%), followed by MICU non-COVID shift participants (48.23%), and MICU COVID shift participants (46%). MICU COVID shift participants spent more time in normal stress level, followed by MICU non-COVID shift participants and CVICU participants. Analysis of differences in physiological variables between COVID and non-COVID participants in MICU (see Table 4) revealed that non-COVID shift participants exhibited significantly higher SI, HR, ST, and EE than COVID shift participants (*p* < 0.001 for all variables). Phasic EDA was higher for COVID participants, but this difference was not statistically significant (*p* = 0.15).

**Table 3:** Percent time spent in high, normal, and low stress levels by the participants.

	High stress level (SI ≥ 12.2) (%)	Normal stress level (SI < 12.2) (%)
CVICU	54.3	45.29
MICU (non-COVID shifts)	48.23	51.76
MICU (COVID shifts)	46	53.98

**Table 4:** Descriptive statistics of physiological variables of MICU participants working COVID and non-COVID shifts.

Physiological variables	COVID shifts	Non-COVID shifts
Stress index, SI (c.u.), *M* (*SD*)	12.24 (2.66)	12.53 (3.09)
Heart rate, HR (bpm), *M* (*SD*)	69.5 (8.26)	70 (8.89)
Skin temp, ST (°C), *M* (*SD*)	32.90 (1.74)	33.31 (1.42)
Phasic EDA (μS), *M* (*SD*)	0.255 (0.57)	0.153 (0.29)
Energy expenditure, EE (kcal/min), *M* (*SD*)	0.95 (1.40)	1.06 (1.49)

However, multiple logistic regression models after adjusting for demographics (age, number of children, and years of nursing experience in the ICU), physiological variables (HR, phasic EDA, ST, EE), and emotional exhaustion showed that the ratio of the probability of experiencing high stress to the probability of not experiencing high stress was 3.71 times higher for COVID unit participants (OR = 3.71; 95% CI [1.87, 7.38]; *p* < 0.001; degrees of freedom [DF] = 26,992) in comparison to non-COVID unit participants. Additionally, we did not find any differences in the stress levels when the same participants had alternate postings in the COVID and non-COVID units (OR = 0.97; 95% CI [0.86, 1.09]; *p* = 0.58; DF = 41,117).

### 3.2. Qualitative findings

The qualitative findings focused on comparing MICU and CVICU participants’ rationale behind their perceived stress during the working shifts. Findings showed seven common themes grouping contributors of stress among both MICU and CVICU participants: patients’ families, patient acuity, clinical procedures, admittance of new patients, proning, laboratory and imaging, and helping coworkers. Out of the seven identified themes, subthemes evolved only for two themes, patient acuity and clinical procedures.

#### 3.2.1. Patient families

Participants mentioned communication/interaction with family members of patients as a major source of stress. M1 (M = MICU participant) noted “family drama with patients’ family” and C3 (C = CVICU participant) mentioned “rude, demanding patient family” as examples of significant stressors.

#### 3.2.2. Admittance of new patients

Participants in both units discussed new patient admissions and incoming patient transfers as stressors.

#### 3.2.3. Proning

Participants stated that proning, a process for safely turning a patient onto their abdomen from their back which requires at least six ICU personnel, is a cause of stress during the shift.

#### 3.2.4. Laboratory and imaging

Collecting samples from patients for lab tests and taking the patient for imaging were mentioned by participants as stressful events during their shift. Participant M14 elaborated:

“Obtaining blood cultures and urine cultures are stressful and very tasky. Nurses need to present the necessity of obtaining cultures to the doctors and charge nurse. Urine cultures are also a hassle because we need to discontinue current Foley catheter and place a new one.”

#### 3.2.5. Helping coworkers

Participants at both units stated that though their shift assignment was not stressful, constantly helping their neighboring nurse or other coworkers in the unit increased their workload and led to significant stress. M13 mentioned “helping colleague with [their] patient,” as major contributor to their stress. Furthermore, death of an adjacent patient instigated stress in nurses. Participant M2 mentioned, “Patient next door got unstable. He was coded twice and died.”

#### 3.2.6. Patient acuity

Patient acuity level, an indicator of the amount of nursing care required, was widely discussed by the participants in both units as a major source of stress. Subthemes emerged as the units cared for different patient populations. Participants at MICU mentioned caring for COVID-positive patients, unstable non-COVID patients, and patients undergoing sudden cardiac arrest as their stressors. For example, M5 mentioned “two COVID-positive patients, both on pressors… both patients on ventilators, no restraints” and M14 cited an “actively dying COVID patient,” emphasizing the influence of caring for COVID patients on their increased stress. Similarly, participants cited “unstable bleeding patient requiring emergency surgery at the start of shift” [M12], and “patient coding” [M2, M6, and M9] to indicate the effect of caring for unstable patients and coding patients on their stress. Participants at CVICU stated caring for critically ill cardiovascular patients as patient acuity factors leading to stress.

#### 3.2.7. Clinical procedures

Participants from both units identified performing specific COVID-related procedures as stressors. MICU participants identified intubating patients as the most stressful event. Additionally, performing continuous renal replacement therapy, a procedure to support renal function for critically ill patients, was mentioned by MICU participants as a stressful procedure. Participants at CVICU cited that bronchoscopy—a procedure to visualize the patient’s airways—as difficult and stressful.

## 4. Discussion

Our study investigated differences in prevalence and causes of occupational stress and burnout among nurses working in COVID and non-COVID ICUs in a large hospital in the Southwestern United States, as well as compared the within-subject effect of caring for COVID and non-COVID patients. Findings from the pre-study questionnaires suggest that the COVID unit participants experience higher occupational stress and burnout than the non-COVID unit participants. Increased emotional exhaustion, anxiety, and perceived stress scores were prevalent among COVID unit participants. High emotional exhaustion, a primary indicator of burnout, is associated with low job satisfaction (57), cognitive withdrawal from their job and organization (58), and reduced quality of life (59). Increased perceived stress contributes to high anxiety and impairs concentration and decision-making of the nurses (60). Constant anxiety eventually leads to fatigue, reduced self-confidence, increased workplace stress, and lowers job performance (61). This is evident by the COVID unit participants exhibiting a low personal achievement score, while the non-COVID unit participants exhibited high level of personal achievement.

Acute fatigue, a consequence of new procedures and work-related events, can be addressed by sufficient recovery/break times (62). Without adequate time to recover, acute fatigue transitions to chronic fatigue which is detrimental to the overall wellbeing of personnel and negatively influences job performance (62). Though our participants in both units presented similar acute fatigue scores, the COVID unit participants had low inter-shift recovery and high chronic fatigue than the non-COVID unit participants. This is concerning, as past studies (63, 64) found that increased work fatigue in nurses negatively influenced task-based aspects of their work and led to diminished individualized nursing care toward patients. A combination of increased emotional exhaustion, perceived stress, anxiety, chronic fatigue, and reduced personal achievement and inter-shift recovery suggests higher level of occupational stress and burnout among nurses working in COVID units than nurses in non-COVID units. This is in line with prior work which demonstrated that the uncertain treatment outcomes of COVID patients and the fear of getting infected induce stress and burnout among nurses working with COVID patients (20).

On the other hand, the findings from physiological indices revealed high HR, ST, and phasic EDA among non-COVID unit participants. Additionally, non-COVID unit participants spent 54% of their shift time in high stress level, and their SI was higher than MICU participants. When comparing within the COVID unit participants who cared for both COVID and non-COVID patients, the physiological indices were higher during non-COVID shifts than the COVID shifts. These findings suggest contradictory accounts of stress and burnout between self-reported measures and objective physiological metrics of this study. One explanation for such non-congruence may be due to the impact of higher physical activity on physiological variables and SI. For instance, SI calculated from the Baevsky’s Stress Index characterizes the activity of sympathetic nervous system which is elevated during the periods of high cognitive stress and physical activity. Similarly, HR, phasic EDA, and skin temperature are also influenced by both cognitive and physical stress (35, 48). This is in line with a recent study (65) which found that the number of steps walked by the nurses while caring for a COVID patient was lower than for a non-COVID patient. The constant need to don and doff the protective wear and the high level of care demanded by COVID patients might have restricted the nurses to stay within the patient room, hence requiring the neighboring nurse working with a non-COVID patient to perform tasks for the COVID nurse. This is manifested by higher energy expenditures of non-COVID shift MICU participants than the COVID shift MICU and CVICU participants. Furthermore, COVID patients needed more bedside monitoring, proning, hemofiltration, and hygiene procedures than non-COVID patients, requiring more nursing time (66, 67). Reduced movements within the unit and relentless attention sought by the COVID patients may have influenced the physiological indices of the COVID-shift participants. These findings may suggest that while physiological variables have shown promise in assessing occupational stress and burnout in recent literature, results from such methods may not be robust-enough to assess cognitive stress and highlight the need to interpret findings from physiological methods in combination with self-reported measures.

To address this, a bivariable and multivariable logistic regression model comparing the physiological stress between MICU COVID-only and CVICU participants while accounting for self-reported metrics such as emotional exhaustion was used and the findings revealed that the participants caring for only COVID patients experienced 3.71 times more stress than CVICU participants who cared exclusively for non-COVID cardiovascular patients. Using the same model, no significant differences in stress levels were found within the MICU participants who worked both COVID and non-COVID shifts. This finding may indicate that despite the potentially lower physical stress demands of care for COVID patients compared to non-COVID patients, the mere presence of COVID patients in the same unit had some influence on the non-COVID patients and the occupational stress and burnout of nurses caring for them. This is further validated by our qualitative findings in which several of the COVID unit participants stated that the high acuity and presence of COVID patients increased their stress and workload. This is in line with prior studies that refer to non-COVID patients as “collateral damages” (68) and discussed the effects of COVID on such patients and the nurses caring for them (17, 69) which may suggest the negative impact on the entire unit regardless of individual assignment to COVID vs. non-COVID patients.

### 4.1. Limitations

This work was conducted at the intersection of Delta and Omicron variants’ predominant period, which may have influenced our study findings. Though the pandemic was still ongoing, most healthcare settings, including our study site, had sufficient resources which may have reduced occupational stress experienced by nurses at the beginning of the pandemic. Another limitation was the relatively low and unequal number of participants from CVICU and MICU. Although the study aimed to recruit an equal number of participants from both units, the ongoing pandemic, Omicron variant, and longitudinal nature of the study affected participants’ commitment to the study. Attrition notwithstanding, data were collected for six 12-h shifts, rendering 4,320 min of physiological data across all participants. Nevertheless, to our knowledge, this is the first study that naturalistically evaluate occupational stress of ICU nurses comparing both COVID and non-COVID using a combination of objective and self-reported metrics. Lastly, this study should be generalized with caution since the data were collected from one hospital and only two ICUs; findings may vary at non-COVID ICUs other than the CVICU studied and other health systems. Burnout is a complex and multifaceted phenomenon including several factors not measured in this study and indeed understudied in general (7) as well as occupation-external stressors that may affect nurses, such as loss of personal loved ones to the pandemic (70). It is therefore important to expand and diversify studies of stress and burnout among nurses in a variety of settings.

## 5. Conclusion

While the impact of the COVID-19 pandemic on nurses’ occupational stress and burnout has been studied, previous research has seldom used objective methods of assessing stress. In a separate study (71), our team conducted focus group interviews with 20 nurses to identify the contributors and practical mitigators of burnout during the COVID-19 pandemic. This study revealed five themes as burnout contributors at systems level with several subthemes under each of them. The nurses also identified several mitigators to burnout. The current work documents the prevalence and causes of occupational stress and burnout among nurses working in COVID and non-COVID ICUs using validated questionnaires, wrist-worn sensors, and open-ended responses. The results showed a disparity between perceived stress/burnout and stress measured using physiological metrics. When these metrics were combined, nurses primarily working with COVID patients were about four time more likely to be stressed than nurses working with non-COVID patients. No significant differences in stress levels were found among nurses who worked with COVID and non-COVID patients at the COVID unit. The overall causes of stress during the shift expressed by COVID and non-COVID unit nurses were similar. These findings indicate that the nurses working in COVID units, irrespective of whether they care for a COVID patient, experience occupational stress and burnout. Self-reported or physiological metrics in isolation may not provide a ‘big picture’ of occupational stress and burnout for ICU nurses, and mixed-methods investigations are necessary to inform strategies to overcome the impact on ICU nurses’ overall wellbeing. Our prior work (71) in combination with the current study informs the implementation of effective strategies that may help the ICU nurses to cope with the pandemic-influenced burnout.

## Data availability statement

The datasets presented in this article are not readily available because data sharing was not included in the approved IRB protocol. Requests to access the datasets should be directed to fsasangohar@houstonmethodist.org.

## Ethics statement

The studies involving human participants were reviewed and approved by Houston Methodist Research Institute IRB. The patients/participants provided their written informed consent to participate in this study.

## Author contributions

FS and FM contributed to conception of the study. PS and FS designed the study. PS, TN, QZ, and FS contributed to the formal analyses of the data. PS worked on original draft preparation. FS, TN, FM, and QZ contributed toward reviewing and critically revising the draft. All authors have read and agreed to the published version of the manuscript.

## Conflict of interest

The authors declare that the research was conducted in the absence of any commercial or financial relationships that could be construed as a potential conflict of interest.

## Publisher’s note

All claims expressed in this article are solely those of the authors and do not necessarily represent those of their affiliated organizations, or those of the publisher, the editors and the reviewers. Any product that may be evaluated in this article, or claim that may be made by its manufacturer, is not guaranteed or endorsed by the publisher.
